# Enabling Factors, Barriers, and Perceptions of Pneumococcal Vaccination Strategy Implementation: A Qualitative Study

**DOI:** 10.3390/vaccines10071164

**Published:** 2022-07-21

**Authors:** Junjie Huang, Fung-Yu Mak, Yuet-Yan Wong, Samantha Ko, Marc K. C. Chong, Zixin Wang, Kam-Lun Hon, Eliza L. Y. Wong, Eng-Kiong Yeoh, Martin C. S. Wong

**Affiliations:** 1 Jockey Club School of Public Health and Primary Care, Chinese University of Hong Kong, Hong Kong 999077, China; junjie_huang@link.cuhk.edu.hk (J.H.); felixmak@cuhk.edu.hk (F.-Y.M.); yuetyanwong@cuhk.edu.hk (Y.-Y.W.); samanthatszwingko@cuhk.edu.hk (S.K.); marc@cuhk.edu.hk (M.K.C.C.); wangzx@cuhk.edu.hk (Z.W.); ehon@hotmail.com (K.-L.H.); lywong@cuhk.edu.hk (E.L.Y.W.); yeoh_ek@cuhk.edu.hk (E.-K.Y.); 2 School of Public Health, Peking Union Medical College, Beijing 100005, China; 3 School of Public Health, Peking University, Beijing 100191, China

**Keywords:** IPD, barriers, facilitators, perceptions, CFIR, REAIM, Hong Kong, qualitative study

## Abstract

Invasive pneumococcal disease (IPD) is a leading cause of disability and mortality worldwide, particularly in the elderly population. With the implementation of the Government Vaccination Programme (GVP) and the Vaccination Subsidy Scheme (VSS), enabling factors and barriers in service provider scheme participation and vaccination uptake were examined in 32 interviews with doctors and 16 interviews with vaccine recipients. Interview data were analysed in NVivo 11.0 with reference to the Consolidated Framework for Implementation Research (CFIR) and the REAIM Framework to develop codes and themes. Barriers to pneumococcal vaccination uptake included concerns on vaccine efficacy and poor understanding of the disease and vaccine schemes, whilst service provider participation was hindered by ill-defined parameters for patient eligibility and time, location, and logistical constraints. Enabling factors to improve intervention implementation were involvement of the government and physicians to encourage participation, clarifying eligibility criteria, and improving individual knowledge of IPD and vaccination schemes. As participation rates in the GVP and VSS remains low in Hong Kong, efforts concentrating on health promotion strategies encouraging pneumococcal vaccination amongst the elderly population are recommended.

## 1. Introduction

Invasive pneumococcal disease (IPD), caused by *Streptococcus pneumoniae*, is a leading cause of disability and mortality worldwide [1,2]; it has a significant public health impact and seriously affects the elderly population, aged 65 years and above, who are at high risk of serious complications and mortality [3,4]. From 2014 to 2018, a strong upward trend was observed in pneumonia cases with pneumonia being the second highest cause of mortality in Hong Kong [5]. From 2007 to 2015, the annual incidence of IPD ranged from 1.7 to 2.9 per 100,000 [6]. The total number of adults in Hong Kong aged 65 and above was over 1.146 million in 2019, accounting for over 15.7% of the total population [7]. Although a decline in IPD was observed following the COVID-19 outbreak, the vaccination coverage in the high-risk category of individuals aged 65 years and above is considered low at 45.8% [8].

The pneumococcal vaccine can greatly lower the risk of pneumococcal illness or IPD in the elderly [9,10]. Pneumococcal vaccine was linked to a substantial reduction in the risk of pneumococcal illness in a retrospective cohort study of 47,365 older people aged 65 and up (hazard ratio, 0.56) [11]. Pneumococcal immunisation may lessen the risk of ischemic stroke and myocardial infarction [12,13]; these observations provide important demographic data for public health policy in childhood immunisation programme [14]; however, uptake rates for pneumococcal vaccination have remained low despite evidence of its effectiveness. In the United States, 61.3% of senior persons had seasonal pneumococcal immunization in 2013–2014 [15]. In England, the pneumococcal vaccine uptake rate for older individuals was 69.8% in 2014–2015 [16]. Only 34% of elderly persons in Hong Kong over the age of 65 have had pneumococcal vaccination [17].

In October 2019, the Hong Kong Special Administrative Region (HKSAR) Government devised two programs—the Vaccination Subsidy Scheme (VSS) and the Government Vaccination Programme (GVP)—to encourage individuals to sign up for vaccination [18,19]. The VSS covers the cost of influenza and pneumonia vaccines in the enrolled private sector for 210 Hong Kong Dollar (HKD) (27 United States Dollar (USD)) and HKD 250 (USD 32). From 2017 and 2018 onwards, the VSS supports pneumococcal infection prevention by subsidising both the 23-valent polysaccharide pneumococcal vaccine (23vPPV) and the 13-valent pneumococcal conjugate vaccination (PCV13). The GVP provides free pneumococcal immunisation to eligible populations in Hospital Authority clinics, Department of Health authorised centres, and aged residential care homes [18]. Adults aged 65 and above, with or without high-risk health conditions, are considered eligible subjects. The GVP and VSS are complex interventions that are best evaluated using frameworks for implementation research [20,21,22,23,24]. The objective of this study was to perform a qualitative study examining factors influencing vaccination uptake and the participation of physicians, policy makers, and vaccine recipients in the schemes.

## 2. Materials and Methods

### 2.1. In-Depth Face-to-Face Interviews for Physicians and Policy-Makers

#### Settings and Subject Recruitment

Doctors from different sectors and policy-makers from the Food and Health Bureau and Department of health were invited to participate in the study. A purposive sampling methodology was employed to recruit six types of service providers. The service provider groups include:

1. Group-practicing doctors working in the private sector, including doctors running group practice or working in Health Maintenance Organizations (HMOs);
2. Primary Care Practitioners (PCPs) working in Non-Government Organizations (NGOs);
3. PCPs who were in solo practice;
4. PCP in District Health Centres or Community Health Centres;
5. Practitioners in Public Care Settings, such as the General Out Patient Clinic (GOPC) under the jurisdiction of the Hospital Authority;
6. Policy-makers from the Food and Health Bureau (FHB) and the Department of Health (DH) who are involved in the formulation and implementation of the GVP/VSS.

As a result, there were a total of 6 distinct groups. Although some of the physicians were not involved in the vaccination programmes, the attitudes and opinions form different specialities of doctors were important to understand a full spectrum of the vaccination programmes. The purpose of dividing them into different subgroups is to increase the generalisability of the participants. Thus, we did not aim to compare the opinions for different subgroups. In-depth interviews were conducted in a designated interview room with each interview lasting approximately 20–30 min. The interview process and study objectives were explained to the subjects prior to the interview. A total of 32 interviews were carried out and the sample sizes were common generally [25]. Four interviewees were recruited for each of the six groups and additional interviews were conducted until data saturation was achieved. A minimum of two interviewees were recruited from non-enrolees for groups (1) to (4). To examine the meaning, process interpretation and theory, an interview guide for doctors and service providers (Supplementary Material S1) was utilised to evaluate the opinions across the different groups, allowing the researchers to gain deeper insight into the phenomenon under investigation.

We applied a combined Consolidated Framework for Implementation Research (CFIR; Supplementary Material S3) [20,21,22,23,24] and the RE-AIM framework (Supplementary Material S4) [26] to evaluate the governmental GVP and VSS programmes alongside the perception of the pneumococcal vaccine. Both frameworks were derived from theories and their joint application included a comprehensive set of implementation determinants and intervention development [22]. The CFIR framework focused on evaluating the process, while the RE-AIM framework examined scheme implementation outcomes. The template for GVP/VSS evaluation was further modified by a panel of experts consisting of epidemiologists, physicians who specialised in infectious diseases, psychologists, and public health professionals assembled at the commencement of the project.

### 2.2. In-Depth Face-to-Face Interviews for Vaccine Recipients

#### Settings and Subject Recruitment

Individuals who had received pneumococcal vaccines through the GVP/VSS were recruited if they: (1) were aged 65 years or above; (2) could communicate in Chinese; and (3) resided in a Hong Kong household at the time of the study.

The sample was made up of purposively sampled residents and participants were grouped according to their gender (male vs. female) and the presence of risk factors for pneumococcal infection. Twelve patient interviews were conducted as a minimum of three interviewees were recruited for each group (male subjects with risk factors; male subjects without risk factors; female subjects with risk factors; female subjects without risk factors). An additional two interviews per group were conducted amongst prospective vaccine recipients who did not receive the vaccine through the GVP/VSS. A total of 16 patients were interviewed. The sample sizes of patients exceeded the minimum number suggested in the previous study [25]. Before commencing the interview, participants completed a survey on their socio-demographic factors. Open-ended questions related to the barriers, facilitators, perceptions, attitudes, and satisfaction of the GVP/VSS were asked in the interview. After the questions in the interview guide (Supplementary Material S2) had been answered, participants had the option to provide additional comments. If important new insights were raised during the second interview in both groups, additional interviews were held until data saturation.

### 2.3. Data Processing and Analysis

Following the thematic analysis approach, all interviews were transcribed verbatim from audio records by the research team following each interview session, and the transcription was double-checked to ensure accuracy. Using the NVivo program (QSR International, Australia), two investigators (CFIR and RE-AIM) coded the transcripts of the interviews to analysis the interview transcripts. A deductive method was used to code the transcripts’ significant words and phrases. Key concepts from the phrases and sentences were condensed into a few concise remarks. Two investigators (CFIR and RE-AIM) examined the interpretation of statements and coding data to reduce researcher bias. A narrative theme was identified for each individual interview. Segments of the transcript were re-analysed in order to summarise the narrative theme. Data were analysed in relation to previously established topic areas (CFIR: Dimension 1: Characteristics of the intervention; Dimension 2: Inner setting; Dimension 3: Outer setting; Dimension 4: Individual involved; Dimension 5: Implementation process. RE-AIM: Dimension 1: Reach; Dimension 2: Effectiveness; Dimension 3: Adoption; Dimension 4: Implementation; Dimension 5: Maintenance.) and any other new categories that surfaced during the interview. Disputes were resolved through discussions regarding the appropriate coding and categorisation of narrative themes. The original language of interview transcripts was Cantonese and the chosen representative quotes were translated into English to aid in the development of survey instruments for future research in subsequent years.

## 3. Results

### 3.1. Physicians and Policy-Makers

The characteristics of service providers can be found in Table 1. The number of codes by theme among service providers was reported in Supplementary Table S1.

#### 3.1.1. Theme 1: Reasons for Service Providers Not to Join the GVP/ VSS Scheme

Service providers were reluctant to join the GVP/VSS scheme due to the perceived specialty of the vaccination programmes, namely the public health or primary care sector. The majority of service providers who did not participate in the vaccination scheme specialised in medical specialities unrelated to primary care and healthcare.

*My specialty is orthopaedics, which is not related to the primary health care…*

*-Group 1, Doctor 3*

Doctors may also avoid participating in the vaccination scheme as involvement would require follow-up consultations which would increase their daily workload:

*It raises my workload, that’s why I don’t join the vaccination schemes.*

*-Group 3, Doctor 12*

#### 3.1.2. Theme 2: Difficulties in Providing Vaccination Service

There were varying opinions received from the service providers regarding the arrangement of the vaccination scheme. Those who perceived the scheme negatively expressed that the scheme was not adequately organised, and it was not promoted widely enough to increase awareness.

Service providers also faced varying difficulties in the provision of the vaccination service. There were insufficient venues or a lack of space for distributing the vaccine. Procedural requirements from the Department of Health complicated the workflow as service providers worked to ensure no mistakes were made when evaluating eligibility criteria of patients. Additionally, no official recording system was available to facilitate communication between the private and public health sectors.

*Manpower is not that difficult to handle, but the main problem is the venues.*

*-Group 6, Doctor 27*

*Sometimes we may not have patient’s record, so sometimes it’s hard to estimate how many people received vaccine before. In fact, there should be more than that. We don’t seem to have an overall record of how many people received vaccine before.*

*-Group 5, Doctor 26*

Some service providers found that the criteria to determine patients’ eligibility for receiving the vaccination was ill-defined. Without clear guidelines, doctors would offer conflicting recommendations—such as whether to receive the PPV13 or PPV23 first—as each service provider may have had a different definition of high-risk groups.

*I think the problem is the design of the program, because you need to meet some conditions to receive PCV13, of course they will have a query to determine whether you meet it or not…people with uncomplicated hypertension may not meet this condition, there are some grey areas…*

*-Group 1, Doctor 2*

Some service providers faced issues with the price and supply of the pneumococcal vaccine. Some stated that the high price deterred patients from getting vaccinated whilst others found that vaccine supply was inconsistent and insufficient. As such, it was difficult to complete the vaccination during influenza season.

*Another problem is actually the source of supply, that is, when pneumococcus vaccine supply is not enough, then pneumococcus vaccine is not my first priority…*

*-Group 2, Doctor 11*

#### 3.1.3. Theme 3: General Public’s Awareness toward the Vaccination Scheme

The general public’s awareness of the vaccination scheme was low as there was a lack of promotion by the Government. Many service providers expressed that majority of the elderly population did not know detailed information about the scheme.

*For the general public and elderly, the recognition and acceptance of the vaccination schemes is not high.*

*-Group 1, Doctor 4*

#### 3.1.4. Theme 4: Impact of COVID-19

Some service providers commented that COVID-19 strengthened motivation for receiving vaccinations; however, the pneumococcal vaccination was mistaken for the COVID-19 vaccine which further complicated promotion and education of the vaccination scheme.

*Another difficulty is due to the COVID19 pandemic. They are afraid of going to the clinic or seeing doctors. Even if I want to encourage the vaccine or educate my patients, there is no chance.*

*-Group 4, Doctor 2*

A group of service providers found that the COVID-19 pandemic was deemed more influential due to its high-risk status, and therefore required urgent attention compared to other diseases that were less invasive with lower incidence rates. Thus, the importance of the pneumococcal vaccine was overlooked.

*Not enough, because all the attention shifted to COVID-19. No one talk about pneumonia now, and the promotion GVP is not enough now.*

*-Group 2, Doctor 8*

#### 3.1.5. Theme 5: Attitude towards the GVP/VSS Schemes of Service Providers

In general, the satisfaction rate amongst vaccine service providers was relatively high; they claimed that the vaccination schemes were effective and helpful to the patients and would like to recommend their patients to join the vaccination schemes; however, vaccination rate could be improved further.

*I think it’s all good, because this plan has been running for many years... so I think that the whole GVP plan is very satisfactory.*

*-Group 5, Doctor 24*

#### 3.1.6. Theme 6: Suggestions to Further Improve of the GVP/VSS Schemes

1. Criteria

Service providers suggested simplifying the criteria of the vaccination scheme and to establish a clearer definition of the recommended age range and high-risk group.

*For example, even if you are over 65 years old, which is called high risk and you can receive PCV13, maybe it can be relaxed in these criteria, so the take up rate will be increase.*

*-Group 1, Doctor 2*

2. Promotion and education for patients

Some service providers indicated that promotion of the vaccine scheme and educational resources were limited for patients. They suggested the Government should endeavour to deliver more information to the general public to effectively improve understanding by resolving patient confusion.

*I think there should have more education...…*

*-Group 2, Doctor 9*

*I have saw the advertisements on TV about pneumococcus, it should tell more information, like data, so that people feel that there is a necessary to receive the pneumococcal vaccine.*

*-Group 4, Doctor 20*

3. Consultation time for service providers and patients

Service providers indicated that consultation time for patients was limited and more educational material should be provided by the Government prior to the consultation; this would ensure a reduction in consultation time needed and ease the workload for service providers.

*It takes me five minutes or ten minutes to explain that the pneumococcal vaccine, it is not really enough (time)…*

*-Group 1, Doctor 4*

### 3.2. Vaccine Recipients

The characteristics of vaccine recipients can be found in Table 2. The number of codes by theme among patients was reported in Supplementary Table S2.

#### 3.2.1. Theme 1: Reason for Patients to Receive/not to Receive Pneumococcal Vaccine

1. To receive pneumococcal vaccine

Healthcare-related factors represent one of the major concerns for receiving the pneumococcal vaccine. Some vaccine recipients expressed that receiving pneumococcal vaccine could prevent pneumococcus.

*I think that the resistance will be weaker when you are older, so I think it is an appropriate time to receive this pneumococcal vaccine injection, which will have a positive protective effect on your body.*

*-Patient 12*

Some patients were encouraged to follow the advice provided by doctors and the Government:

*My husband and I went to a private hospital to see a doctor, and I saw the government’s propaganda posters, and it said the criteria age, and I reach the age, then I will participate in this vaccination program.*

*-Patient 13*

2. Not to receive pneumococcal vaccine

The most significant reasons for patients who did not receive the pneumococcal vaccine was the lack of trust in its effectiveness and safety or due to their lack of knowledge on the vaccine.

*I heard that if you receive this vaccine, you will die. Later, I heard that your face would be paralysed after receiving this vaccine. I felt stressed about it...*

*-Patient 8*

#### 3.2.2. Theme 2: Patients’ Satisfaction in the GVP/VSS

Vaccine recipients felt comfortable throughout the vaccination process and viewed their experience of the vaccination procedures positively in relation to its arrangement, appointment reservation, and accessibility of vaccination sites.

*It’s convenient. I don’t need to make an appointment or wait for the doctor to be able to receive it. After the injection, they will tell you anything you should pay attention to. It’s comfortable to me, I think.*

*-Patient 3*

#### 3.2.3. Theme 3: Instructions Comments of Pre- and Post-Vaccination Given by Clinic Doctors or Staff

Vaccine recipients commented that the instructions given by doctors and clinical staff prior to receiving the vaccination and after were insufficient. Only a few sentences were asked, such as “How do you feel after receiving the vaccine?” and “The side effects are common. Do not worry.” After the injection, there were no other comments or instructions provided to vaccine recipients.

*They just simply explained that we will send your medical history to doctors and look at it, so if a doctor doesn’t have any special comment, then I can make an appointment to receive the vaccine.*

*-Patient 2*

#### 3.2.4. Theme 4: Suggestions to Further Improve the Vaccination Arrangement

1. Promotion

The interviewees suggested more promotion from public hospitals and comprehensive explanations from doctors could be viable strategies in promoting the GVP and VSS scheme.

*I think the government has to take the initiative, the government is so passive... Government should require private doctors, as well as doctor stations, nurse stations, etc., to encourage more people to receive the vaccine.*

*-Patient 14*

Additionally, it would be beneficial to utilise more materials, such as leaflets, posters, television programmes, radio broadcasts, and social media. Promotional campaign should target the elderly with detailed information defining high-risk groups who are especially vulnerable.

*The poster needs to be in-depth and clearly. The elderly really likes to go to the elderly centers. Then more posters should be provided to the elderly centers.*

*-Patient 1*

*Usually, many elderly people like to watch TV shows. It is better to strengthen the propaganda on TV shows. So, when the elderly see it, they will know there are vaccination subsidy program for them.*

*-Patient 13*

2. Criteria of GPV/VSS

To increase the vaccination rate of the pneumococcal vaccine, criteria for eligible recipients should be relaxed so that more people can participate in the vaccination scheme.

*(So, you think that it’s better to remove the age boundary) Sure.*

*-Patients 15 and 16*

## 4. Discussion

### 4.1. Major Findings

Through the interviews conducted, factors and attitudes affecting medical service providers and vaccine recipients’ participation in the Government vaccination schemes were examined. Doctors and healthcare specialists who expressed unwillingness to participate in the schemes cited the following reasons: (1) vaccinations are irrelevant to the field of healthcare services they provide; (2) participation would increase their daily work-load; (3) insufficient consultation time to educate patients effectively. Although a high satisfaction rate amongst vaccine service providers was reported, common attitudes towards difficulties encountered include: (4) lack of venues available for distributing the vaccine; (5) no official recording system available; (6) ill-defined criteria for determining high-risk individuals and patient eligibility to participate in the scheme; (7) inconsistent supply and vaccine cost; (8) COVID-19 vaccine as mistaken for the PV vaccine. For vaccine recipients, factors affecting scheme participation and vaccination intention or hesitation were: (9) preventing pneumococcal infection; (10) encouragement from doctors and the Government; (11) safety concerns; (12) lack of knowledge on the vaccination. Vaccine recipients al-so were relatively satisfied with the scheme arrangements but found that: (13) insufficient medical advice and instruction was provided from medical staff before and after receiving the vaccine; (14) strict eligibility criteria made the scheme less accessible.

### 4.2. Explanations and Literature Review

From 2007 to September 2015, 70% and 81% of IPD cases in adults 65 years of age and older were caused by serotypes covered by PCV13 and 23vPPV respectively. Although 23vPPV contains 11 additional serotypes and theoretically offers extra protection, Immunogenicity studies on PCV13 and 23vPPV showed that PCV13 elicited non- inferior or better immune response for serotypes commonly covered by both vaccines [27]. In other words, the PCV13 can provided enough protection to the current Hong Kong population. In general, the GVP/VSS schemes covered both PCV13 and 23vPPV; however, there was difference in the amount of subsidy between PCV13 and 23vPPV. The amount of subsidy for one dose of PCV13 was higher than that for one dose of 23vPPV in the schemes. Improving patient knowledge and awareness of the vaccination schemes and the pneumococcal vaccine can help increase vaccination rates [28,29]; moreover, healthcare knowledge is a decisive factor in influence patient decision-making and health recommendations from trusted medical professionals may strengthen patient confidence [30,31]. The difficulties experienced by both service providers and vaccine recipients may have a significant effect on the vaccination rate as vaccine intention may of-ten be impacted by a myriad of difficulties [28,32,33]. The COVID-19 pandemic has caused unprecedented disease burden upon the healthcare system and has affected accessibility to medical services treating other condition and illnesses [34]. Fear of contracting COVID-19 may have led to vaccine hesitation and prevented individuals from visiting private clinics and hospitals to receive the pneumococcal vaccine, thus leading to reduced participation in the schemes and lower vaccination rates. Expanding the age groups to 60 years old or younger age groups as well as improving the vaccine coverage of the 65 years old population are important to increase the total vaccine uptake rate in the Hong Kong population. Enhancing knowledge of vaccine and awareness of the schemes amongst older age groups may raise motivation to receive the vaccine and improve overall vaccination rates.

### 4.3. Limitations

Some limitations of the study should be mentioned. Firstly, the data were obtained from interviews, thus resulting in a small sample size. Secondly, the survey only included participants from the Chinese population, therefore the findings’ generalizability may be limited. As the study was limited to participants who were over 65 years of age due to the stringent requirements necessary to participate in the vaccination scheme, the data obtained may not have accurately represented the experience of the general elderly population, especially individuals <65 years old who had yet to be vaccinated; moreover, it is difficult to replicate results as participant responses are subjective. There may be varying differences in their personal experiences, which limits the transferability of results as it may not be applicable to all medical service providers and elderly patients. To corroborate our findings, prospective studies involving more ethnic groups and an examination of a larger sample size is required; moreover, children’s pneumococcal vaccination strategies were very important. The overall coverage of vaccine uptake rate was high. Our study did not look into the pneumococcal vaccination for children which is also important due to its close relationship with elderly infection and disease; however, the overall coverage of PCV vaccine uptake rate was high among children in Hong Kong. From 2012–2014, the uptake rate of 3–5 years old children who had completed 4 dose PCV was up to 95% [35]. Therefore, we focused on the investigation of pneumococcal vaccination in the elderly in the current study.

### 4.4. Implications

To ensure the success of GVP and VSS schemes, it is important to increase the coverage and participation of the target population as much as possible. The vaccination uptake rate is a critical determinant for the reduction of pneumococcus incidence and mortality at the population level. Findings of the present study identified the enabling factors, barriers, perceptions of the pneumococcal vaccination, attitudes towards the GVP/VSS schemes, and formulation and implementation of the schemes. The result suggests that redefining the criteria would help in clarifying the confusions among doctors and general public. The Government should provide a feedback process to collect opinions from the public to establish a clearer framework for eligibility criteria. The recommendations from participating doctors and patients in Study 2 suggested increasing promotion and educating the public would help propagate the vaccination schemes. The dissemination of educational materials for all age groups should provide a comprehensive explanation of the Government vaccination schemes. Information could be delivered through regular workshops in community centres, broadcasts, banners, leaflets, advertisements, and promotion on social media. The strategies could explore the effectiveness of disseminating information to different social groups, such as young generation, to improve vaccination rates in the future.

As the current study is exploratory, it opens up many possibilities for future research both in terms of theory building and concept validation. To corroborate the results of this study, it may be beneficial to explore factors influencing pneumococcal vaccination intention using approaches based on different behavioural theories. Future research could extend the sample size to include a larger portion of the population such as targeting the younger population and examining their attitudes towards the vaccine to extract a more comprehensive set of data. As the COVID-19 pandemic was a newly observed factor that influenced vaccination intention, future studies could assess the impact of COVID-19 upon the public’s attitude towards vaccines.

## 5. Conclusions

To increase the uptake rate of pneumococcal vaccination, the Government should increase its efforts in encouraging participation in the vaccination schemes offered by the Hong Kong Government. The Government and clinicians should work together to promote the GVP and VSS by boosting its perceived benefits and the self-efficacy of pneumococcal vaccination, whilst lowering the barriers and difficulties for achieving immunisation among the elderly.

## Figures and Tables

**Table 1:** General characteristics of physicians and policy-makers.

Participants Codes	Group	Categories	Role of Participation
1	Group 1	HMOs	Service provider
2	Group 1	HMOs	Service provider
3	Group 1	HMOs	Not participating
4	Group 1	HMOs	Service provider
5	Group 1	HMOs	Not participating
6	Group 1	HMOs	Service provider
7	Group 2	PCPs in NGOs	Service provider
8	Group 2	PCPs in NGOs	Not participating
9	Group 2	PCPs in NGOs	Service provider
10	Group 2	PCPs in NGOs	Service provider
11	Group 2	PCPs in NGOs	Service provider
12	Group 2	PCPs in NGOs	Not participating
13	Group 3	PCPs in solo practice	Not participating
14	Group 3	PCPs in solo practice	Not participating
15	Group 3	PCPs in solo practice	Service provider
16	Group 3	PCPs in solo practice	Service provider
17	Group 3	PCPs in solo practice	Service provider
18	Group 3	PCPs in solo practice	Service provider
19	Group 4	PCP in DHC/CHC	Service provider
20	Group 4	PCP in DHC/CHC	Not participating
21	Group 4	PCP in DHC/CHC	Not participating
22	Group 4	PCP in DHC/CHC	Service provider
23	Group 4	PCP in DHC/CHC	Service provider
24	Group 4	PCP in DHC/CHC	Service provider
25	Group 5	GOPC under HA	Service provider
26	Group 5	GOPC under HA	Service provider
27	Group 5	Policy-makers from FHB and DH	Service provider
28	Group 5	Policy-makers from FHB and DH	Policy maker
29	Group 6	Policy-makers from FHB and DH	Policy maker
30	Group 6	Policy-makers from FHB and DH	Policy maker
31	Group 6	Policy-makers from FHB and DH	Policy maker
32	Group 6	Policy-makers from FHB and DH	Policy maker

**Table 2:** General characteristics of vaccine recipients.

Participants Codes	Gender	Risk Factors	Uptake Vaccine
1	Female	No	No
2	Female	High	No
3	Male	High	Yes
4	Male	High	Yes
5	Female	High	Yes
6	Female	High	Yes
7	Male	High	No
8	Male	No	No
9	Male	High	Yes
10	Female	High	Yes
11	Female	No	Yes
12	Female	No	Yes
13	Female	No	Yes
14	Female	No	Yes
15	Male	No	Yes
16	Male	No	Yes

## Data Availability

Please contact the corresponding author for more information.

## Supplementary Materials

The following supporting information can be downloaded at https://www.mdpi.com/article/10.3390/vaccines10071164/s1, Supplementary Material S1: Interview guide of doctors and services providers. Supplementary Material S2: Interview guide of vaccine recipients. Supplementary Material S3: Consolidated Framework for Implementation Research (CFIR). Supplementary Material S4: RE-AIM (Reach, Effectiveness, Adoption, Implementation, Maintenance) Framework. Table S1: Number of codes by theme among service providers. Table S2: Number of codes by theme among patients.
