# Supplementary material for: Enabling Factors, Barriers, and Perceptions of Pneumococcal Vaccination Strategy Implementation: A Qualitative Study
Source: Vaccines (Basel). 2022 Jul 21;10(7):1164. doi: 10.3390/vaccines10071164 (PMC9315707; doi:10.3390/vaccines10071164)
Supplement: Supplementary file 1 [file vaccines-10-01164-s001.zip › vaccines-1783578-supplementary.pdf]

Supplementary materials

Supplementary material S1 Interview guide of doctors and services providers

Supplementary material S2 Interview guide of vaccine recipients

Supplementary material S3 Consolidated Framework for Implementation Research (CFIR)

Supplementary material S4 RE-AIM (Reach, Effectiveness, Adoption, Implementation, Maintenance) Framework

Supplementary table S1 Number of codes by theme among service providers Supplementary table S2 Number of codes by theme among patients

Supplementary material S1. Interview guide of doctors and services providers

1. **Observations from service providers**
   a. As a service provider of the vaccination scheme, what do you think about the arrangements of the scheme? What difficulties have you encountered?
   b. Before joining this programme, have you ever provided vaccination service? In your experience, are there any recommended practices for vaccination?
   c. Does participating in this program increase your workload by a lot? Do you think the consultation time provided for the participants is sufficient?

2. Observations regarding the general public
   a. In your opinion, how much do citizens aged over 65 value and know about vaccination?
   b. Do you think the government's work in promotion of this vaccination scheme and recruitment of participants is sufficient? Do you have any other suggestions?

3. Observations regarding participants
   a. What obstacles and difficulties do you think the participants have when participating in this vaccination programme?
   b. Is the participants' compliance good after participating in the vaccination scheme? Do you have any suggestions for improvement?
   c. During the whole process, what do you most hope participants can collaborate in doing?
   d. Do you think this plan benefits the health of the participants?
   e. How satisfied are the participants with the vaccination scheme? Did they provide any feedback to the doctor?
   f. Do you think the participants and doctors trust each other? Is there any problem with communication?

4. Observations regarding the vaccine plan
   a. Do you think the procedures in the vaccination scheme, such as inspection, consultation, participant education, and e.t.c., are appropriate? Is there any suggestions for improvement?
   b. Overall, are you satisfied with the current vaccination scheme? Is this plan effective, convenient and feasible?
   c. Would you recommend this vaccination scheme to your patients (who have not participated yet)?
   d. In your opinion, in terms of epidemic prevention, what other practical approaches can the government adopt in addition to vaccination?

5. Recommendations of Government Vaccination Programme (GVP) or Vaccination Subsidy Scheme (VSS)
   a. Would you recommend this programme to your family and friends?

Supplementary material S2. Interview guide of patients

1. Awareness of vaccination as a means of prevention for Pneumococcal infection
    a. Is it your first time receiving Pneumococcal vaccination?
        i. If yes, is there any reason for not receiving Pneumococcal vaccination before?
        ii. If no, why did you receive Pneumococcal vaccination before?
    b. Did you notice any promotion of Government Vaccination Programme (GVP) or Vaccination Subsidy Scheme (VSS) from the Government?
        i. If yes, can you list any channels?
    c. Do you have any suggestions to improve the promotion of Government Vaccination Programme (GVP) or Vaccination Subsidy Scheme (VSS)?

2. Knowledge about pneumococcus and the pneumococcal vaccination
    a. What do you know about pneumococcus?
        i. What are the ways to prevent Pneumococcal infection?
    b. What is pneumococcal vaccination?
        i. Who should receive pneumococcal vaccination?
        ii. What are the risk factors of Pneumococcal infection?
        iii. What do you think your risk of acquiring Pneumococcal infection is?
        iv. Why do you think your risk is high/ average?

3. Attitude and perception towards pneumococcal vaccination
    a. How would you describe your attitude towards pneumococcal vaccination programme in general?
    b. What motivated you to enrol in Government Vaccination Programme (GVP) or Vaccination Subsidy Scheme (VSS)?
    c. Did you encounter any barriers when enrolling in the programme?
    d. What are the difficulties you experienced when going through the Government Vaccination Programme (GVP) or Vaccination Subsidy Scheme (VSS)?
    e. What are some positive points and do you have any suggestions for improvement?

4. Satisfaction of Government Vaccination Programme (GVP) or Vaccination Subsidy Scheme (VSS)?
    a. Can you share with us your experience of going through the vaccination process? Any particular feelings and comments?
        i. Were the arrangements of Government Vaccination Programme (GVP) or Vaccination Subsidy Scheme (VSS) clearly explained to you by clinic doctor or their staff?
        ii. Were the details of the processes involved in Government Vaccination Programme (GVP) or Vaccination Subsidy Scheme (VSS) clearly explained to you by clinic doctor or their staff?
        iii. Can you list a few instructions given by clinic doctor or their staff about pneumococcal vaccination?

b. (If applicable) Has the doctor clearly explained the details and risks of the entire pneumococcal vaccination procedure?
    i. Could you recall the risks of pneumococcal vaccination which were told by clinical doctor or their staff?
    ii. Do you have any suggestions to improve the pneumococcal vaccination arrangement?

c. Did you read carefully the programme materials such as guidelines, leaflets and posters? Do you have any suggestions?
    i. Are they clear, practical and easy to be understood?

5. Practice in Government Vaccination Programme (GVP) or Vaccination Subsidy Scheme (VSS)
    a. Would you recommend this programme to your family and friends?

Supplementary material S3. Consolidated Framework for Implementation Research (CFIR)

Dimension 1: Characteristics of the intervention[20-24]

Construct 1: Intervention source

Perception of key stakeholders about whether the vaccination programmes are externally or internally developed (by interviews of policy-makers)

Construct 2: Evidence strength and quality

Indicator: Evidence basis for GVP/VSS in enhancing pneumococcal vaccine uptake (i.e. why did policy makers believe such programmes are able to increase vaccine uptake; how did policy makers determine the amount of Government subsidy; what threshold level of quality did the programme consider as robust enough to adopt and translate into policy?) (by interviews of policy makers and review of programme documentations)

Construct 3: Adaptability

Indicator: The degree to which the GVP/VSS can be adapted, tailored, refined, or reinvented to meet patent needs in the population (by interviews of policy-makers and PCPs)

Construct 4: Cost

Indicator: The costs of formulation of the GVP/VSS and the costs associated with implementing the vaccine programmes, including investment, supply, and opportunity costs (by interviews of policymakers).

Dimension 2: Inner setting

Construct 1: Structural characteristics

Indicator: 1) whether there are designated staff in the clinic who are in charge of providing pneumococcal vaccine (by interviews of the clinics-in-charge)

Construct 2: Readiness for implementation

Indicator: 1) whether there is sufficient staff to carry out the vaccine under GVP/VSS, 2) whether there is a protocol for screening patients and administering the vaccine under GVP/VSS, 3) whether there is sufficient storage of PCV (by interviews of the clinic-in-charge)

Dimension 3: Outer setting

Construct 1: Patients needs and resources

Indicator: 1) whether Primary Care Practitioners (PCPs) in designated public clinics are aware of patients' concerns and barriers for taking up the vaccine (in-depth interview of the PCPs)

Dimension 4: Individual involved

Construct 1: Knowledge and beliefs about the intervention

Indicator: 1) PCPs in designated public clinics are aware of the GVP/VSS for pneumococcal vaccines; 2) belief (perceived benefits and barriers for implementing GVP/VSS) (in-depth interview of the PCPs)

Construct 2: Self-efficacy

Indicator: 1) confidence of implementing GVP/VSS of pneumococcal vaccine in the population (in-depth interview of the PCPs)

Construct 3: Individual stage of change (overlap with Adoption of the REAIM framework, please see below)

Dimension 5: Implementation process

Construct 1: Engaging appropriate individuals in the implementation

Indicator: 1) What stakeholders did policy makers identify for implementing the vaccines and the rationale behind (by interviews of policy-makers)

Construct 2: Reflecting and evaluating

Indicator: 1) Whether policy-makers monitor the implementation of GVP/VSS (number of vaccine uptake each year, coverage of vaccines in the target population), and 2) whether policy makers make adjustment/refinement of GVP/VSS based on the monitoring results. (by interviews of policy-makers)

Supplementary material S4. RE-AIM (Reach**, E**ffectiveness**, A**doption**, I**mplementation**, M**aintenance**) framework** [20-24]

Dimension 1: Reach

Meaning: number, proportion and representativeness of eligible elderly individuals who have participated in the GVP/VSS; IndicatorI: 1) Percentage of elderly who had heard of GVP/VSS for vaccination (by population-based survey), 2) Whether elderly individuals were aware of the arrangements of the GVP/VSS (cost, who can receive free vaccines, location for vaccination, procedure to make appointment for vaccination) (by population-based survey)

Dimension 2: Effectiveness

Meaning: intervention effect on targeted outcomes

Indicator: 1) uptake rate of vaccine (by population-based survey), 2) use of GVP/VSS to receive pneumococcal vaccine instead of elsewhere (by population-based survey)

Dimension 3: Adoption

Meaning: number, percentage and representativeness of participating service providers

Indicator: 1) Whether PCPs in designated public clinics have introduced the vaccine under GVP/VSS to eligible patients (by interviews of PCPs)

2) Whether PCPs in designated public clinics provide pneumococcal vaccines under GVP/VSS to eligible
patients (by interviews of PCPs)

3) Any extra cost when pneumococcal vaccines are provided under VSS for PCPs (by interviews of PCPs)

Dimension 4: Implementation

Meaning: The extent to which the intervention was consistently implemented by service providers

Indicator: 1) The extent to which PCPs in designated public clinics follow the protocol of providing pneumococcal vaccines under the GVP/VSS (by interviews of PCPs, or make use of standard patients)

Dimension 5: Maintenance

Meaning: the extent to which an intervention becomes part of routine organizational practices

Indicator: 1) Whether providing pneumococcal vaccines under GVP/VSS is a part of routine service in private/designated public clinics (by interviews of clinic-in-charge)

Supplementary table S1. Number of codes by theme among service providers

Themes	Coding no.	No. of codes
reason for not participating_specialist	1	7
reason for not participating_workload	2	5
the arrangements of the scheme	3	16
difficulties in providing vaccination service_no	4	6
difficulties in providing vaccination service_spacing	5	7
difficulties in providing vaccination service_procedures	6	14
difficulties in providing vaccination service_criteria&subsidy	7	34
difficulties in providing vaccination service_identify necessity	8	1
difficulties in providing vaccination service_homosexuality	9	1
difficulties in providing vaccination service_record	10	12
difficulties in providing vaccination service_supply of vaccine	11	10
resources_manpower	12	16
resources_financing (payment method, additional fee)	13	9
resources_equipment (education materials, information for patient)	14	17
experience of providing vaccination service	15	34
recommended practices for vaccination	16	44
workload	17	26
consultation time provided for participants	18	36
value and knowledge of elderly about the vaccination (citizens who aged over 65)	19	34
awareness of the public	20	15
government's work_promotion	21	103
government's work_recruitment of participants	22	9
government's work_policy-making	23	11
difficulties of the participants_knowledge	24	32
difficulties of the participants_belief	25	7
difficulties of the participants_feelings	26	19
difficulties of the participants_decision making	27	5
difficulties of the participants_cost	28	10
difficulties of the participants_no	29	1
compliance of the participants	30	44
collaboration of participants	31	27
benefits of the participants' health	32	26
satisfication of the participants	33	23
feedback from the participants	34	14
communication between doctors and participants	35	14
trust between doctors and participants	36	28

**procedures_eligibility checking**	37	37
**procedures_consultation**	38	2
**procedures_education**	39	12
**procedures_suggestions for improvement**	40	31
**overall satisficiation of the doctors**	41	25
**satisficiation of the doctors_effective**	42	12
**satisficiation of the doctors_convenient**	43	3
**satisficiation of the doctors_feasible**	44	7
**cannot continue**	45	3
**continue**	46	6
**benefits of the doctor**	47	1
**communication among doctors**	48	3
**epidemic prevention_other practical approaches**	49	30
**recommend to the patients**	50	30
**recommend to family and friends**	51	25
**eHealth_access to information**	52	6
**COVID19**	53	41

Supplementary table S2. Number of codes by theme among patients

Themes	Coding no.	No. of codes
received pneumococcal vaccination before	1	25
reason for receiving pneumococcal vaccination before	2	15
not received pneumococcal vaccination before	3	4
reason for not receiving pneumococcal vaccination before	4	12
notification of government promotion on gvp/vss	5	25
channels of promotion	6	51
suggestions of government promotion	7	49
knowledge about pneumococcus	8	10
knowledge about pneumococcus_no	9	7
ways to prevent pneumococcal infection	10	20
knowledge about pneumococcal vaccination	11	7
knowledge about pneumococcal vaccination_no	12	7
targets for pneumococcal vaccination	13	19
risk factors of pneumococcal infection	14	21
personal risk of acquiring pneumococcal infection	15	19
reason for the perception of personal risk	16	18
attitude towards pneumococcal vaccination programme	17	31
attitude against pneumococcal vaccination programme	18	7
motivation to enroll in gvp/vss	19	23
barriers when enrolling in the programme	20	16
difficulties when participating in the programme	21	27
positive points/improvement	22	27
personal experience/ satisfaction of going through the vaccination process	23	10
explanation of the programme arrangements by clinic doctor or their staff	24	17
explanation of the programme processes by clinic doctor or their staff	25	9
instructions given by clinic doctor or their staff	26	12
explanation of the details and risks of the vaccination	27	21
the risks of pneumococcal vaccination that have been explained by doctors	28	11
improvement of the vaccination arrangement	29	21
programme materials	30	23
improvement of the programme materials	31	10
clear, practical and easy to be understood	32	8
recommend to family and friends	33	26
covid19	34	15
flu vaccination	35	15
